# Failure to reduce the risk of postoperative lower genital tract infection with perioperative antibiotic prophylaxis during induced abortion: a real-world study

**DOI:** 10.3389/fmed.2024.1296910

**Published:** 2024-02-08

**Authors:** Jing Ding, Yan Zhang, XiangYing Gu, Yan Che

**Affiliations:** ^1^NHC Key Lab of Reproduction Regulation, Shanghai Engineering Research Center of Reproductive Health Drug and Devices, Shanghai Institute for Biomedical and Pharmaceutical Technologies, Shanghai, China; ^2^State Key Laboratory of Genetic Engineering, School of Life Sciences, Fudan University, Shanghai, China; ^3^General Hospital of Tianjin Medical University, Tianjin, China

**Keywords:** induced abortion, antibiotics, lower genital tract infection, risk, perioperative period

## Abstract

**Objective:**

This study aimed to evaluate perioperative antibiotic use for induced abortion and its association with lower genital tract infections (LGTI) two weeks post-surgery in China.

**Methods:**

We conducted a prospective cohort study of women seeking induced abortion. We interviewed participants on the day of surgery and two weeks after, and evaluated preoperative tests, gynecological exams, perioperative antibiotic usage, postoperative vaginal microbiota, and LGTI diagnosis. Multivariate logistic regression was used to assess the association between the perioperative antibiotic use and LGTI risk.

**Results:**

We recruited 8,190 women undergoing induced abortion at 27 participating hospitals. Of these, 95% had gynecological exams, but over 80% lacked tests for vaginal microbiota, chlamydia, and gonorrhea. Approximately 20% of those examined had increased vaginal discharge and abnormal vaginal cleanliness. The positive rates for gram-positive rods, fungi, and trichomonas were 38.6, 2.4, and 0.3%, respectively. More than three-quarters (78.5%) of participants received antibiotics, mainly second-gen cephalosporins (36.8%) and nitroimidazoles (12.3%). LGTI rates two weeks post-surgery were 2.7% for antibiotic recipients and 3.1% for non-recipients, with no statistically significant difference (*p* > 0.05). Logistic regression showed no association between perioperative antibiotic use and LGTI risk (OR = 1.01, 95% CI 0.59–1.74). However, this risk increased with abnormal preoperative discharge tests (OR = 1.39, 95% CI 1.04–1.86).

**Conclusion:**

Most Chinese women undergoing induced abortion used perioperative antibiotics, but this did not significantly reduce LGTI risk. Instead, this risk was related to abnormal preoperative discharge tests. Standardization of perioperative antibiotic use for induced abortion is recommended, and prophylactic treatment in Chinese abortion services warrants further investigation.

## Highlights

- This study aims to address the knowledge gap regarding the practical effectiveness of perioperative antibiotic prophylaxis in induced abortion surgeries.
- This study challenges the efficacy of perioperative antibiotic prophylaxis during induced abortion in reducing postoperative lower genital tract infections, shedding light on potential reconsiderations of current medical practices.

## 1 Introduction

Induced abortion is a legal procedure in China and is commonly used as a back-up or therapeutic method to terminate pregnancies due to unwanted or pathological pregnancies (1). The number of induced abortions in China in recent decades has been between 9 and 10 million per year, and the incidence of abortion was approximately 28.88 per 1,000 women of reproductive age in 2019 (2). Previous studies have shown that induced abortion may result in pain, bleeding, incomplete abortion, lower genital tract infection (LGTI) and other complications (3), and the risk of LGTI increases with the number of abortions (4). LGTI refers to infections that occur in the reproductive organs located in the lower part of the female reproductive system, such as the vagina and cervix (5), and was a common complication of induced abortion (6). It has been reported that the rate of infections requiring antimicrobial treatment after early induced abortion ranges from 0.01 to 2.44% (6). The presence of preoperative LGTI not only delays the optimal timing of treatment but also increases the risk of postoperative tubal adhesions, cervical adhesions, tubal obstruction, and chronic pelvic pain, as well as the risk of ectopic pregnancy, spontaneous abortion, placental adhesion, or implantation, thus significantly compromising women’s reproductive health (7). The World Health Organization’s “Safe Abortion: Technical and Policy Guidance for Health Systems” (8) recommends the prophylactic use of antibiotics to reduce the risk of infection following abortion. The American College of Obstetricians and Gynecologists (6) recommends the prophylactic use of antibiotics, preferably doxycycline, nitroimidazoles and tetracyclines, for all women undergoing a surgical abortion, but acknowledges the need to consider potential side effects. The Society of Obstetricians and Gynecologists of Canada (9) recommends the preoperative use of antibiotics, including doxycycline, metronidazole, and beta-lactams, for all women undergoing surgical abortion. These options are commonly used, and none is superior to others. The “Chinese Expert Consensus on the Prophylactic Use of Antibiotics in Induced Abortion (2019)” (10) and the “Chinese Expert Consensus on Screening, Diagnosis, and Treatment of LGTI during the Perioperative Period of Induced Abortion (2022)” (11) also recommend the prophylactic use of antibiotics, including doxycycline 200 mg, minocycline 200 mg, azithromycin 500 mg, or metronidazole 1 g before surgical abortion. However, there are few clinical studies evaluating the effectiveness of prophylactic use of antibiotics for induced abortion in China. We therefore conducted this multicentre prospective cohort study to understand the status of preoperative laboratory and gynecological examinations and perioperative antibiotic use in induced abortion and their association with LGTI, with the aim of providing evidence for clinical practice in abortion services in China.

## 2 Materials and methods

### 2.1 Study sites

A total, 27 hospitals with outpatient induced abortion services were selected from 16 provinces in the eastern (Fujian, Shandong, Shanghai, Tianjin, Zhejiang), central (Guangxi, Jilin, Jiangxi, Shanxi) and western (Inner Mongolia, Guizhou, Shaanxi, Xinjiang, Yunnan, Sichuan, Chongqing) regions of China. Each institution recruited women who met the inclusion criteria for induced abortion on a continuous basis for two consecutive months in 2019.

### 2.2 Inclusion and exclusion criteria and study methods

We conducted this prospective observational cohort study, which was approved by The Ethics Committee of the Shanghai Institute of Planned Parenthood research (IRB number: PJ2019-22). Women seeking an induced abortion at the study sites were invited to participate in this study. Inclusion criteria for participants were as follows: women aged 18 years or older, intrauterine pregnancy confirmed by ultrasound, voluntary acceptance of induced abortion In early pregnancy (<10 gestational weeks), no contraindications to surgical abortion, being able to understand and answer survey questions, being willing to participate in this study and sign an informed consent form, and being willing to undergo follow-up when returning for a medical examination two weeks after surgery. Patients were excluded if they had undergone an in-patient abortion, medical abortion, spontaneous abortion, or other pathological abortion, if they had a serious chronic disease, or if the physician deemed them inappropriate to participate in this study.

Participants underwent routine pre-and post-operative examinations, and laboratory tests at the hospital. Data on the examinations and tests were extracted from the hospital’s electronic medical record, including temperature, pulse, and relevant gynecological examinations such as the cervix, uterus, and adnexa; detection of vaginal microbiota morphology such as vaginal cleanliness, discharge, and microbial flora; the metabolic products of aerobic bacteria, anaerobic bacteria, fungi, trichomonas, and pH value, etc. Information on antibiotic use was collected at the follow-up interviews two weeks after surgery, including the name of the antibiotics, use dosage, medication regimen, and duration of use. The diagnosis of LGTI was extracted from participants’ medical records, which was diagnosed mainly based on associated symptoms (itching or burning of the vulva), and lab examination of vaginal discharge. We added up the abnormal results of all the laboratory tests and defined a new variable to measure the number of abnormal vaginal discharge tests.

### 2.3 Statistical methods

We used EpiData 3.1 for data entry and R 4.0.2 software for data analysis. We used frequencies and percentages to describe the distribution of categorical data, and the chi-squared test to compare the difference in the distribution between categories of baseline characteristics. In addition, a two-level multivariate logistic regression model was used to control for potential confounders and to examine the effectiveness of perioperative antibiotic use on LGTI at two weeks postabortion. A *p*-value of less than 0.05 was considered statistically significant.

## 3 Results

### 3.1 Baseline characteristics of the study participants

A total of 8,190 women who underwent induced abortion were recruited for this study. Most participants were from tertiary hospitals (84.7%) and maternal and child hospitals (MCHs) (56.4%). The average age of the participants was 29.69 ± 5.98 years, with the majority aged 26–35 years (55.7%). Most participants (72.1%) were married, 40.4% had a bachelor’s degree or higher education level, and 22.5 and 38.4% were employed in manual and intellectual labor, respectively. The majority (73.9%) had their first sexual experience between the ages of 18 and 25 years. The majority (91.9%) reported no sexual intercourse during menstruation, and 59.8% had a history of gynecological and/or obstetric infectious diseases. (Table 1).

**Table 1:** Baseline characteristics of study participants.

Characteristics	*n* (%) (*N* = 8,190)	Characteristics	*n* (%) (*N* = 8,190)
Hospital-level		Education level	
Secondary	1,254 (15.3)	Middle School or below	1,415 (17.5)
Tertiary	6,936 (84.7)	High School/ College	3,401 (42.1)
Hospital-type		Bachelor’s Degree or above	3,261 (40.4)
Maternal and child hospital	4,618 (56.4)	Occupation	
General Hospital	3,573 (43.6)	Physical Labor	1774 (22.5)
Age (years)		Mental Labor	3,036 (38.4)
≤26	2,217 (27.2)	Other	3,090 (39.1)
26 ~ 30	2,396 (29.3)	Age of Sexual Debut (years)	
31 ~ 35	2078 (25.5)	<18	1,148 (14.7)
>35	1,464 (18.0)	18–20	2,229 (28.6)
Marital Status		21–25	3,536 (45.3)
Married	5,758 (72.1)	>25	890 (11.4)
Unmarried	2,228 (27.9)	Sexual intercourse during menstruation	
History of gynecological infection		Yes	650 (8.1)
Vaginitis	3,504 (43.7)	No	7,417 (91.9)
Pelvic inflammatory disease	1,291 (16.1)		
No	3,226 (40.2)		

### 3.2 Gynecological examination and preoperative vaginal microbiota

As presented in Table 2, the majority of participants had routine gynecological examinations. For example, uterine mobility and tenderness, cervical tenderness, lower abdominal tenderness, pelvic examination, and perianal examination were reported in over 95% of women, and body temperature, cervical smear, and external genitalia were reported more than 80% of participants. Abnormal findings are generally low, most ranging from 0.2 to 0.6%, with a few over 2%, and cervical erosion (21.3%) and cervical smear color (8.4%) being the most common abnormalities.

**Table 2:** Routine gynecological examinations and vaginal microecological laboratory tests for women seeking abortion services.

Examination/laboratory test	Frequency and proportion (%) of participants having examination/test (*N* = 8,190)	Frequency and proportion (%) of abnormal results
*Routine gynecological examination*		
Uterine mobility	8,115 (99.1)	19 (0.2)
Uterine tenderness	8,115 (99.1)	16 (0.2)
Right adnexal examination	8,105 (99.0)	33 (0.4)
Left adnexal examination	8,095 (98.8)	26 (0.3)
Cervical tenderness	8,069 (98.5)	22 (0.3)
Perianal examination	7,930 (96.8)	48 (0.6)
Lower abdominal tenderness	7,911 (96.6)	43 (0.5)
Vaginal wall	7,817 (95.4)	160 (2.0)
Cervical erosion	7,794 (95.2)	1,657 (21.3)
Cervical orifice	7,657 (93.5)	215 (2.8)
Body temperature	6,869 (83.9)	104 (1.5)
Cervical smear color	6,673 (81.5)	558 (8.4)
External genitalia	6,658 (81.3)	162 (2.4)
*Vaginal microecological morphological examination*		
Normal flora		
Gram-positive cocci	2085 (25.5)	356 (17.1)
Gram-positive rods	1,398 (17.1)	540 (38.6)
*Pathogens*		
Fungal spores	7,633 (93.2)	187 (2.4)
Fungal hyphae	7,386 (90.2)	136 (1.8)
Gram-negative cocci	2050 (25.0)	335 (16.3)
Gram-negative rods	1816 (22.2)	120 (6.6)
*Chlamydia trachomatis*	1,337 (16.3)	249 (18.6)
*Vaginal microecological function testing*		
Vaginal pH value	7,754 (94.7)	1752 (22.6)
Vaginal secretion odor	7,680 (93.8)	198 (2.6)
Trimethylamine test	2,759 (33.7)	32 (1.2)

Proportion of abnormal result = (number of abnormal cases/number of participants who had the examination or test)* 100.

Testing for vaginal microecological morphology varied between normal flora and potential pathogens. A quarter or less had been tested for gram-positive cocci (25.5%) and rod (17.1%), with positive rates of 17.1 and 38.6%, respectively. A similar proportion of women had been tested for gram-negative cocci (25.0%) and rods (22.2%) and *Chlamydia trachomatis* (16.3%), with positive rates of 16.3, 6.6 and 18.6%, respectively. More than 90% had fungal spore and hyphae testing, of which 2.4 and 1.8% were positive, respectively.

Trimethylamine, vaginal secretion odor and pH value are indicators of vaginal microecological function. One third of the women had a trimethylamine test, and about 94% had vaginal secretion and PH value tests, with positive rates of 1.2, 2.6 and 22.6%, respectively.

### 3.3 Perioperative antibiotic use

Of all the study participants, 78.5% (6,427) used antibiotics in the perioperative period, and all the antibiotics were included in the Chinese guideline. The use of antibiotics was significantly higher in second-level MCHs (96.0%, 1,899/2,318) than in tertiary general hospitals (81.9%, 1,899/2,318), tertiary MCHs (75.0%, 3,462/4,618), and second-level general hospitals (74.2%, 470/633) (*p* < 0.001). The most commonly used antibiotics were second-generation cephalosporins (36.8%, 2,367), followed by nitroimidazoles (12.3%, 788), macrolides (7.5%, 484), tetracyclines (4.9%, 312), and lincomycin (3.4%, 220). Only 51 women (0.8%) used quinolones. In addition, 126 women (2.2%) used antifungals. Of all the antibiotic users, 4,297 (66.9%) used only one type of antibiotic, most commonly the second-generation cephalosporins (37.1%, 1,593); 964 (15.0%) used two or three types of antibiotics, with more than half (482) combining nitroimidazoles and second-generation cephalosporins (Table 3).

**Table 3:** Perioperative antibiotic use in women undergoing induced abortion in China [*n* (%)].

Antibiotics	*n* (%)
*Medication (N = 6,427)*	
Second-generation Cephalosporins	2,367 (36.8)
Nitroimidazoles	788 (12.3)
Macrolides	484 (7.5)
Tetracyclines	312 (4.9)
Lincomycin	220 (3.4)
Antifungals	126 (2.2)
Quinolones	51 (0.8)
*Monotherapy (N = 4,297)*	
Second-generation Cephalosporins	1,593 (37.1)
Macrolides	371 (8.3)
Lincomycin	200 (4.7)
Nitroimidazoles	167 (3.9)
Antifungals	63 (1.5)
Quinolones	49 (1.1)
Tetracyclines	20 (0.5)
*Combination therapy (N = 964)*	
Nitroimidazoles	464 (48.1)
Second-generation Cephalosporins	459 (47.6)
Tetracyclines	264 (27.4)
Macrolides	51 (5.3)
Antifungals	43 (4.5)
Lincomycin	12 (1.2)

### 3.4 Association of laboratory test and perioperative antibiotic use with LGTI

Table 4 shows the incidence of LGTI by women’s characteristics. As can be seen in the table, the incidence of LGTI in women who used antibiotics and those who did not was 2.7 and 3.1%, respectively. The result of the chi-square test shows no significant difference in the rate between the two groups (*p* = 0.513). In addition, we used a two-level logistic regression model adjusting for hospital factors, women’s demographics, and perioperative variables, and found no significant difference in the risk of LGTI two weeks post-induced abortion between those using antibiotics and those not using them (OR = 1.01, 95% CI 0.59–1.74). However, the risk increased with the number of abnormal discharge tests (OR = 1.39, 95% CI 1.04–1.86). No significant associations were observed with hospital factors, women’s demographics, and age at first sexual intercourse (Table 4).

**Table 4:** Lower genital tract infection (LGTI) two weeks after abortion: incidence by women’s characteristics and results of two-level logistic regression analysis.

Characteristics	Number of women	% of LGTI	*Χ*^2^, *p*-value	OR	95% CI
Age (years)			4.505, 0.212		
<26	2,169	3.0		1	
26 ~ 30	2,354	3.1		1.61	0.99–2.60
31 ~ 35	2021	2.8		1.003	0.72–1.48
>35	1,430	2.0		1.15	0.77–1.60
Age of sexual debut (years)			6.141, 0.189		
<18	1,132	2.1		1	
18–20	2,184	2.8		0.765	0.40–1.45
21–25	3,437	3.2		0.685	0.42–1.12
>25	874	2.3		0.585	0.36–0.94
Hospital type			19.215, *P* < 0.001		
Tier3 general hospital	2,225	3.1		1	
Tier3 maternal and child health hospital	4,532	3.1		1.89	0.32–11.09
Tier2 general hospital	633	0.3		1.25	0.05–28.78
Tier2 maternal and child health hospital	619	1.8		1.75	0.73–42.33
Marital status			3.436, 0.179		
Married	5,633	2.6		1	
Unmarried	2,177	3.3		0.93	0.61–1.43
Education			18.841, 0.004		
Middle school and below	1,395	3.6		1	
High school/college	3,319	5.9		1.22	0.26–5.76
Undergraduate and postgraduate	2,728	6.1		2.04	0.44–9.49
Occupation			6.534, 0.366		
Physical labor	1732	6,7		1	
Mental labor	4,427	7.6		2.35	0.87–6.41
Perioperative antibiotic use			0.428, 0.513		
No	999	3.1		1	
Yes	7,010	2.7		1.01	0.59–1.74
Number of abnormal laboratory tests (mean, SD)	1.03, 0.165			1.39	1.04–1.86

## 4 Discussion

### 4.1 Vaginal microbiota assessment before induced abortion

The surgical procedure of induced abortion is considered to be a clean-contaminated procedure, as it causes a degree of trauma to the female uterine cavity, which can disrupt the normal barrier function of the cervix (10). For this reason, routine screening for LGTI is usually required before induced abortion. This study found that the majority (>90%) of patients in China underwent routine vaginal secretion tests and gynecological examinations before abortion. Although these tests and examinations were inexpensive and easily accessible for general screening purposes, they may not detect specific pathogenic microorganisms. More than 70% of women undergoing induced abortion in this study were not tested for potentially pathogenic microorganisms in the vagina, such as gram-negative/positive cocci/bacteria, mycoplasma, and chlamydia. This posed a higher risk of missed diagnoses, as pathogenic microorganisms may enter the uterine cavity through the cervix during the invasive procedure, leading to complications such as uterine and pelvic infections, and, in the long term, tubal obstruction (11). We recommend an increase in testing for vaginal pathogenic microorganisms prior to an induced abortion surgery to facilitate timely intervention and to reduce the risk of LGTI following the procedure.

### 4.2 Conditional vaginal microbiota assessment before induced abortion

Findings of this study showed that abnormal amount of vaginal discharge and abnormal vaginal cleanliness were most frequent in Chinese women seeking abortion services, accounting for about one-fifth of participants who had a vaginal examination. These results were similar to those reported by Sun and colleagues in China (19.6%) (12). The results of the vaginal microbiota testing showed that the prevalence of gram-positive cocci and bacilli, and gram-negative cocci and bacilli was 38.6, 17.1, 16.3 and 6.6%, respectively, and the prevalence of fungi and trichomonas was 2.4 and 0.3%, respectively. These findings suggest that a significant number of Chinese women undergoing induced abortion were positive for conditional vaginal pathogenic microorganisms. Providers should pay due attention to screening for vaginal pathogens before performing abortion.

### 4.3 Antibiotic use during the perioperative period of induced abortion

Approximately four out of five (78.5%) of women undergoing induced abortion received prophylactic antibiotic treatment in the perioperative period. Either the international guidelines [i.e., the World Health Organization’s “Safe Abortion: Technical and Policy Guidance for Health Systems” (8) and the American College of Obstetricians and Gynecologists’ “SFP guideline 20,102” (6)] or the national expert consensus [i.e., the “Chinese Expert Consensus on the Prophylactic Use of Antibiotics in Induced Abortion (2019)” (10)] recommend the prophylactic use of antibiotics before induced abortion surgery. However, a significant proportion of service provides in China did not follow these recommendations. Unexpectedly, the antibiotic use rate was lower in general hospitals and tertiary hospitals than in MCHs and secondary hospitals. This may be due to the differences in evaluation regulations between hospitals in China regarding the antibiotic use. According to the “Circular on Further Strengthening the Management of Antimicrobial Drugs to Curb Drug Resistance “issued by the Ministry of Health (13), the use of antimicrobial drugs is included as an indicator in the evaluation of hospital level and clinical performance. The top-to-bottom hospital evaluation regulation may need be revised to allow the use of antibiotics in abortion services in all levels and types of hospitals to avoid conflicts between clinical need and hospital evaluation regulations.

Previous studies have shown that the pathogens of LGTI after induced abortion are mainly *Escherichia coli*, *Staphylococcus aureus*, and sexually transmitted pathogens such as *Neisseria gonorrhoeae* and *Chlamydia trachomatis* (6). The “Chinese Expert Consensus on the Prophylactic Use of Antibiotics in Induced Abortion (2019)” (10) suggests that prophylactic antibiotics should be effective against aerobic bacteria, anaerobic bacteria, and sexually transmitted disease pathogens. Second-generation cephalosporins, metronidazole, doxycycline, and azithromycin are recommended for first-line use. In this study, we found that the most commonly used antibiotics during the perioperative period of induced abortion in China were second-generation cephalosporins (36.8%), which are effective against gram-positive cocci and gram-negative bacilli. Nitroimidazoles was the second most used antibiotics (12.3%). Previous literature reports that anaerobic spore bacteria are common in postoperative infections after gynecological surgery, and nitroimidazoles are the preferred drugs for the treatment and prevention of anaerobic bacterial infections and trichomoniasis (14). Macrolides accounted for 7.5%, tetracyclines 4.8% and quinolones 0.8% of the antibiotics used. Azithromycin, tetracycline, and quinolones (e.g., levofloxacin) are mainly used to treat *Chlamydia trachomatis* and Mycoplasma dentalium (14). In the group of women who received two or more types of antibiotics, more than half of them used both nitroimidazoles and second-generation cephalosporins. This combination is one of the drug regimens recommended by the Chinese expert consensus (10).

### 4.4 Effectiveness of perioperative antibiotic use on LGTI two weeks postabortion

Furthermore, with an increase in the number of induced abortion procedures, the risk of genital tract infection increases correspondingly (11). This study found that the incidence of LGTI was lower in women who used antibiotics in the perioperative period (2.7%) than that in non-users (3.1%), but the difference was not statistically significant. This conclusion remained after controlling for potential confounders using a two-level multivariate logistic regression model. The main reason for this may be that this study was conducted in a real-world setting and did not establish standardized protocols for antibiotic use and diagnosis of LGTI. The lack of standardization may have led to a certain degree of misclassification and under-diagnosis of LGTI, thereby reducing the effectiveness of antibiotic prophylaxis. Nevertheless, we found that the more positive tests in the preoperative vaginal discharge, the higher the risk of postoperative LGTI (OR = 1.39, 95% CI 1.04–1.86). For those with more than one abnormal vaginal discharge test, we recommend testing for vaginal pathogenic microorganisms and administering antibiotics accordingly.

In summary, about four out of five women who had an induced abortion in China took antibiotics in the perioperative period. However, the use of antibiotics did not significantly reduce the risk of LGTI two weeks after abortion. Instead, the risk was significantly associated with the number of abnormal preoperative vaginal discharge tests. We recommend that the use of antibiotics in the perioperative period of induced abortion should be further standardized. In addition, the effectiveness of prophylactic treatment in the abortion service in China needs further study.

### 4.5 Strengths and limitations

This study was conducted in 27 hospitals in 16 out of 31 provinces in mainland China with a sample size of 8,190. To our knowledge, this is the largest study compared with previous similar studies in China. However, it should be noted that this is an observational study conducted in a real-world setting. The diagnostic of LGTI and laboratory testing methods may vary between hospitals, leading to potential information bias in the results.

## Data availability statement

The original contributions presented in the study are included in the article/supplementary materials, further inquiries can be directed to the corresponding authors.

## Ethics statement

The studies involving humans were approved by the Medical Ethical Committee, Shanghai Institute of Planned Parenthood Research. The studies were conducted in accordance with the local legislation and institutional requirements. The participants provided their written informed consent to participate in this study.

## Author contributions

JD: Writing – original draft. YZ: Data curation, Writing – review & editing. XG: Writing – review & editing. YC: Funding acquisition, Writing – review & editing.
